# 3-(9*H*-Fluoren-9-yl)-1,3-diphenyl­propan-1-one

**DOI:** 10.1107/S1600536812032497

**Published:** 2012-09-05

**Authors:** Fu Feng, Zhi-cai Cui, Xin-ping Liu, Wei-Bing Hu

**Affiliations:** a School of Chemical and Environmental Engineering, Hubei University for Nationalities, Enshi, Hubei 445000, Peoples’ Republic of China

## Abstract

In the title compound, C_28_H_22_O, the fluorene ring system is approximately planar [maximum deviation = 0.044 (2) Å] and forms dihedral angles of 69.88 (6) and 89.46 (6)° with the phenyl rings. The crystal packing is stabilized by weak π–π stacking inter­actions, with centroid–centroid distances of 3.7172 (13) and 3.7827 (11) Å.

## Related literature

For the structure of fluorene, see: Gerkin *et al.* (1984 ▶). For background to the electronic properties of copolymers of poly(alkyl­fluorene), see: Kreyenschmidt *et al.* (1998 ▶). For a description of the Cambridge Structural Database, see: Allen (2002 ▶).
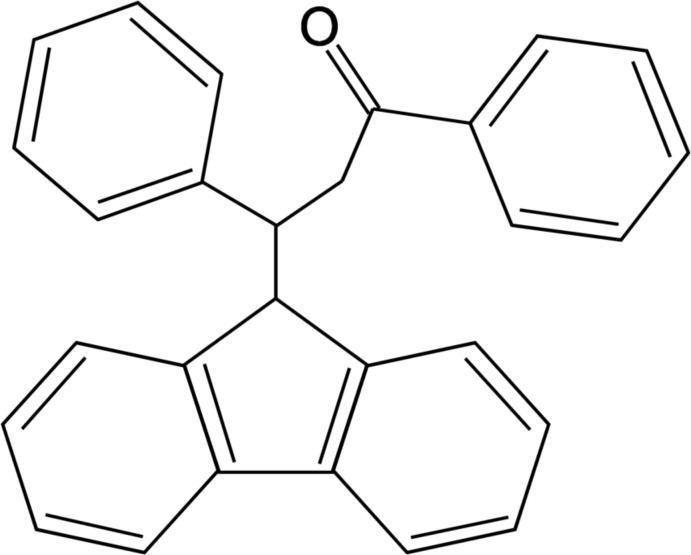


## Experimental

### 

#### Crystal data

C_28_H_22_O
*M* *_r_* = 374.46
Monoclinic, 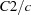

*a* = 15.2433 (5) Å
*b* = 18.3109 (6) Å
*c* = 14.7468 (5) Å
β = 95.708 (6)°
*V* = 4095.7 (2) Å^3^
*Z* = 8
Mo *K*α radiation
μ = 0.07 mm^−1^
*T* = 298 K
0.16 × 0.15 × 0.10 mm

#### Data collection

Bruker SMART APEX CCD area-detector diffractometer
13271 measured reflections
5048 independent reflections
4128 reflections with *I* > 2σ(*I*)
*R*_int_ = 0.023

#### Refinement

*R*[*F* ^2^ > 2σ(*F* ^2^)] = 0.076
*wR*(*F* ^2^) = 0.162
*S* = 1.22
5048 reflections
262 parameters
H-atom parameters constrained
Δρ_max_ = 0.22 e Å^−3^
Δρ_min_ = −0.22 e Å^−3^

### 

Data collection: *APEX2* (Bruker, 2004 ▶); cell refinement: *SAINT* (Bruker, 2004 ▶); data reduction: *SAINT*; program(s) used to solve structure: *SHELXS97* (Sheldrick, 2008 ▶); program(s) used to refine structure: *SHELXL97* (Sheldrick, 2008 ▶); molecular graphics: *SHELXTL* (Sheldrick, 2008 ▶); software used to prepare material for publication: *SHELXTL*.

## Supplementary Material

Crystal structure: contains datablock(s) global, I. DOI: 10.1107/S1600536812032497/rz2788sup1.cif

Structure factors: contains datablock(s) I. DOI: 10.1107/S1600536812032497/rz2788Isup2.hkl

Supplementary material file. DOI: 10.1107/S1600536812032497/rz2788Isup3.cml

Additional supplementary materials:  crystallographic information; 3D view; checkCIF report

## Figures and Tables

**Fig. 1.**
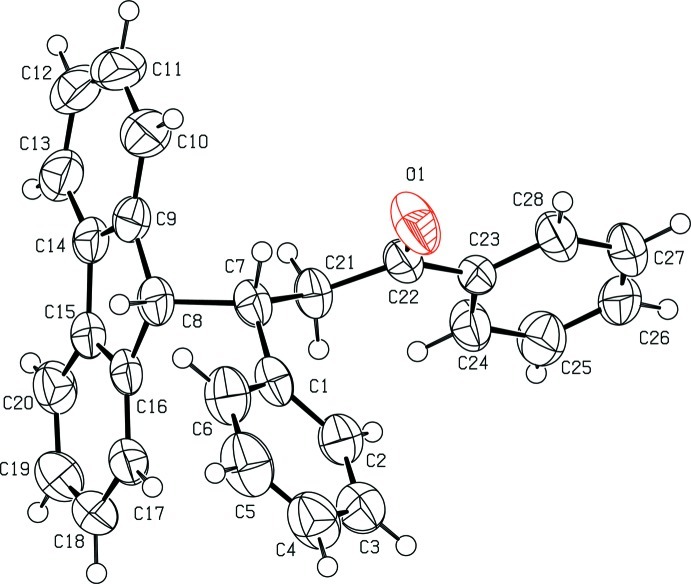
The molecular structure of the title compound, showing 50% probability displacement ellipsoids.

**Fig. 2.**
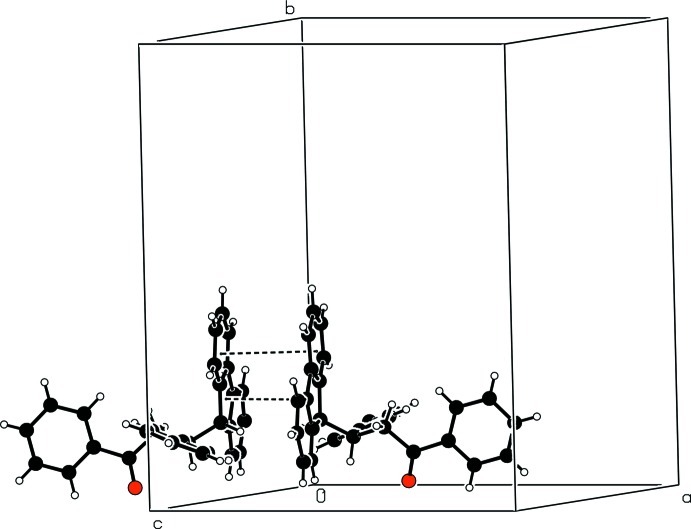
Partial packing diagram of the title compound showing the π–π stacking interactions as dashed lines.

## supplementary crystallographic information

### Comment

Since the publication of its solid-state structure (Gerkin *et al.*, 1984), fluorene and its derivatives have received considerable attention due to their good optical properties and high luminescent efficiencies (Kreyenschmidt *et al.*, 1998). To our knowledge, up to now 2150 structures of fluorene derivatives have been deposited at the Cambridge Crystallographic Data Center (CSD, Version 5.33 of November 2011, plus one update; Allen, 2002). As a contribution to this research field, the structure of the title compound is reported herein.

In the tile compound (Fig. 1), the three fused rings of the fluorene ring system are essentially coplanar, with a maximum deviation from the planarity of 0.044 (2) Å for atom C11. The fluorene moiety forms dihedral angles of 69.88 (6) and 89.46 (6)° with the C1–C6 and C23–C28 phenyl rings, respectively. In the crystal, molecules interact through weak π–π stacking interactions (Fig. 2): Cg1···Cg1^i^, 3.7827(119 Å; Cg2···Cg2^i^, 3.7172 (13) Å (Cg1 and Cg2 are the centroids of the C8/C9C14–C16 and C15–C20 rings; symmetry code: (i) -x, y, 1/2-z). There exist neither classical nor non-classical hydrogen bonds.

### Experimental

Fluorene (2 mmol), chalcone (2 mmol) and NaOH (4 mmol) were mixed in mortar, and the mixture was ground at room temperature for 20 min. The mixture was then washed in sequence with 15 ml aqueous solution of HCl (3%) and ethanol (95%), and the crude product was isolated by filtration. The filtrate was purified by recrystallization from anhydrous ethanol to give the title compound as colourless crystals in 77% yield. Suitable crystals for X-ray analysis were obtained by slow evaporation of a methanol solution at room temperature (m.p. 400–402 K). IR (KBr, ν cm^-1^): 3055, 3021, 2893, 1668, 1596, 1450, 1316, 1232, 1009, 830, 746, 685; ^1^H NMR (DMSO-d_6_, δ): 6.92–8.03 (m, 18H); 4.01 (d, 1H, J = 4.2 Hz), 3.64 (m, 1H), 3.42 (d, 2H, J = 7.0 Hz). Elemental analysis calculated for C_28_H_22_O: C 89.84, H5.88%; found: C 89.97, H 5.71%.

### Refinement

All H atoms were placed in geometrically idealized positions and constrained to ride on their parent atoms, with C—H = 0.93 Å for phenyl H atoms, C—H = 0.97 Å for methylene H atoms, C—H= 0.98 Å for methylidyne H atoms and *U*_iso_(H) = 1.2*U*_eq_(C).

### Crystal data

**Table:**

C_28_H_22_O	*F*(000) = 1584
*M**_r_* = 374.46	*D*_x_ = 1.215 Mg m^−^^3^
Monoclinic, *C*2/*c*	Melting point = 400–402 K
Hall symbol: -C 2yc	Mo *K*α radiation, λ = 0.71073 Å
*a* = 15.2433 (5) Å	Cell parameters from 3982 reflections
*b* = 18.3109 (6) Å	θ = 2.3–28.1°
*c* = 14.7468 (5) Å	µ = 0.07 mm^−^^1^
β = 95.708 (6)°	*T* = 298 K
*V* = 4095.7 (2) Å^3^	Block, colourless
*Z* = 8	0.16 × 0.15 × 0.10 mm

### Data collection

**Table:**

Bruker SMART APEX CCD area-detector diffractometer	4128 reflections with *I* > 2σ(*I*)
Radiation source: fine-focus sealed tube	*R*_int_ = 0.023
Graphite monochromator	θ_max_ = 28.4°, θ_min_ = 2.1°
phi and ω scans	*h* = −19→20
13271 measured reflections	*k* = −22→24
5048 independent reflections	*l* = −18→19

### Refinement

**Table:**

Refinement on *F*^2^	Primary atom site location: structure-invariant direct methods
Least-squares matrix: full	Secondary atom site location: difference Fourier map
*R*[*F*^2^ > 2σ(*F*^2^)] = 0.076	Hydrogen site location: inferred from neighbouring sites
*wR*(*F*^2^) = 0.162	H-atom parameters constrained
*S* = 1.22	*w* = 1/[σ^2^(*F*_o_^2^) + (0.0477*P*)^2^ + 2.3194*P*] where *P* = (*F*_o_^2^ + 2*F*_c_^2^)/3
5048 reflections	(Δ/σ)_max_ = 0.004
262 parameters	Δρ_max_ = 0.22 e Å^−^^3^
0 restraints	Δρ_min_ = −0.22 e Å^−^^3^

### Special details

**Table:**

Geometry. All e.s.d.'s (except the e.s.d. in the dihedral angle between two l.s. planes) are estimated using the full covariance matrix. The cell e.s.d.'s are taken into account individually in the estimation of e.s.d.'s in distances, angles and torsion angles; correlations between e.s.d.'s in cell parameters are only used when they are defined by crystal symmetry. An approximate (isotropic) treatment of cell e.s.d.'s is used for estimating e.s.d.'s involving l.s. planes.
Refinement. Refinement of *F*^2^ against ALL reflections. The weighted *R*-factor *wR* and goodness of fit *S* are based on *F*^2^, conventional *R*-factors *R* are based on *F*, with *F* set to zero for negative *F*^2^. The threshold expression of *F*^2^ > σ(*F*^2^) is used only for calculating *R*-factors(gt) *etc*. and is not relevant to the choice of reflections for refinement. *R*-factors based on *F*^2^ are statistically about twice as large as those based on *F*, and *R*- factors based on ALL data will be even larger.

### Fractional atomic coordinates and isotropic or equivalent isotropic displacement parameters (Å^2^)

**Table:**

	*x*	*y*	*z*	*U*_iso_*/*U*_eq_	
C1	0.14569 (12)	0.11851 (10)	0.03405 (13)	0.0459 (4)	
C2	0.20036 (14)	0.14528 (11)	−0.02784 (15)	0.0559 (5)	
H2	0.2567	0.1611	−0.0068	0.067*	
C3	0.17366 (16)	0.14913 (14)	−0.11970 (16)	0.0683 (6)	
H3	0.2120	0.1669	−0.1598	0.082*	
C4	0.09067 (18)	0.12685 (15)	−0.15199 (18)	0.0751 (7)	
H4	0.0724	0.1294	−0.2140	0.090*	
C5	0.03446 (16)	0.10059 (15)	−0.09216 (18)	0.0758 (7)	
H5	−0.0221	0.0857	−0.1138	0.091*	
C6	0.06160 (14)	0.09625 (12)	−0.00046 (16)	0.0617 (6)	
H6	0.0231	0.0781	0.0392	0.074*	
C7	0.17433 (11)	0.11080 (10)	0.13490 (13)	0.0443 (4)	
H7	0.1705	0.0586	0.1489	0.053*	
C8	0.11128 (12)	0.15036 (10)	0.19542 (13)	0.0467 (4)	
H8	0.0513	0.1323	0.1786	0.056*	
C9	0.13425 (12)	0.13692 (11)	0.29630 (14)	0.0490 (5)	
C10	0.14903 (15)	0.07194 (13)	0.34257 (18)	0.0657 (6)	
H10	0.1446	0.0277	0.3114	0.079*	
C11	0.17051 (16)	0.07338 (16)	0.43584 (19)	0.0774 (8)	
H11	0.1819	0.0299	0.4673	0.093*	
C12	0.17513 (15)	0.13817 (17)	0.48230 (17)	0.0743 (7)	
H12	0.1884	0.1380	0.5452	0.089*	
C13	0.16042 (14)	0.20393 (14)	0.43720 (15)	0.0628 (6)	
H13	0.1633	0.2478	0.4691	0.075*	
C14	0.14135 (12)	0.20290 (11)	0.34337 (13)	0.0488 (5)	
C15	0.12700 (12)	0.26283 (11)	0.27773 (13)	0.0475 (4)	
C16	0.10951 (11)	0.23314 (10)	0.19053 (13)	0.0444 (4)	
C17	0.09007 (13)	0.27879 (11)	0.11672 (14)	0.0545 (5)	
H17	0.0769	0.2596	0.0586	0.065*	
C18	0.09050 (16)	0.35316 (13)	0.13054 (16)	0.0665 (6)	
H18	0.0776	0.3842	0.0811	0.080*	
C19	0.10963 (17)	0.38238 (12)	0.21625 (17)	0.0688 (6)	
H19	0.1104	0.4328	0.2238	0.083*	
C20	0.12763 (15)	0.33779 (12)	0.29069 (15)	0.0602 (6)	
H20	0.1400	0.3575	0.3487	0.072*	
C21	0.27009 (11)	0.13279 (11)	0.16106 (13)	0.0475 (4)	
H21A	0.2808	0.1796	0.1333	0.057*	
H21B	0.2793	0.1390	0.2266	0.057*	
C22	0.33620 (12)	0.07789 (11)	0.13221 (13)	0.0470 (4)	
C23	0.42934 (12)	0.10156 (10)	0.12556 (12)	0.0444 (4)	
C24	0.45648 (13)	0.17306 (12)	0.13258 (15)	0.0571 (5)	
H24	0.4163	0.2092	0.1442	0.069*	
C25	0.54291 (15)	0.19192 (14)	0.12254 (18)	0.0692 (6)	
H25	0.5603	0.2406	0.1272	0.083*	
C26	0.60281 (14)	0.13910 (15)	0.10580 (16)	0.0684 (7)	
H26	0.6609	0.1518	0.0989	0.082*	
C27	0.57709 (16)	0.06803 (15)	0.09925 (19)	0.0764 (7)	
H27	0.6179	0.0321	0.0881	0.092*	
C28	0.49115 (14)	0.04871 (12)	0.10894 (17)	0.0641 (6)	
H28	0.4744	−0.0001	0.1043	0.077*	
O1	0.31441 (10)	0.01523 (8)	0.11616 (13)	0.0772 (5)	

### Atomic displacement parameters (Å^2^)

**Table:**

	*U*^11^	*U*^22^	*U*^33^	*U*^12^	*U*^13^	*U*^23^
C1	0.0368 (10)	0.0394 (9)	0.0612 (12)	0.0054 (7)	0.0038 (8)	−0.0116 (8)
C2	0.0440 (11)	0.0609 (12)	0.0624 (13)	0.0019 (9)	0.0031 (9)	−0.0031 (10)
C3	0.0618 (15)	0.0774 (16)	0.0656 (15)	0.0123 (12)	0.0064 (11)	0.0042 (12)
C4	0.0691 (16)	0.0899 (18)	0.0632 (15)	0.0204 (14)	−0.0096 (12)	−0.0109 (13)
C5	0.0489 (13)	0.0919 (18)	0.0825 (18)	0.0019 (12)	−0.0142 (12)	−0.0216 (14)
C6	0.0426 (11)	0.0694 (14)	0.0725 (15)	−0.0037 (10)	0.0017 (10)	−0.0144 (11)
C7	0.0325 (9)	0.0396 (9)	0.0611 (12)	0.0010 (7)	0.0053 (8)	−0.0051 (8)
C8	0.0288 (9)	0.0538 (11)	0.0584 (12)	−0.0011 (7)	0.0084 (8)	−0.0029 (9)
C9	0.0323 (9)	0.0560 (11)	0.0605 (12)	−0.0021 (8)	0.0138 (8)	0.0075 (9)
C10	0.0542 (13)	0.0599 (13)	0.0847 (17)	−0.0082 (10)	0.0153 (11)	0.0182 (12)
C11	0.0576 (15)	0.0874 (19)	0.0884 (19)	−0.0026 (13)	0.0135 (13)	0.0446 (16)
C12	0.0523 (13)	0.113 (2)	0.0582 (14)	−0.0074 (13)	0.0100 (10)	0.0270 (15)
C13	0.0485 (12)	0.0865 (16)	0.0547 (13)	0.0009 (11)	0.0118 (9)	0.0044 (11)
C14	0.0324 (9)	0.0639 (12)	0.0513 (11)	0.0042 (8)	0.0113 (8)	0.0042 (9)
C15	0.0355 (10)	0.0562 (11)	0.0524 (11)	0.0085 (8)	0.0118 (8)	0.0001 (9)
C16	0.0298 (9)	0.0524 (10)	0.0523 (11)	0.0091 (7)	0.0107 (7)	−0.0012 (8)
C17	0.0509 (12)	0.0629 (13)	0.0507 (11)	0.0182 (10)	0.0097 (9)	0.0007 (9)
C18	0.0736 (16)	0.0621 (13)	0.0660 (14)	0.0266 (11)	0.0177 (12)	0.0148 (11)
C19	0.0804 (17)	0.0478 (12)	0.0800 (16)	0.0189 (11)	0.0173 (13)	0.0013 (11)
C20	0.0613 (14)	0.0602 (13)	0.0600 (13)	0.0120 (10)	0.0107 (10)	−0.0109 (10)
C21	0.0330 (9)	0.0545 (11)	0.0548 (11)	0.0029 (8)	0.0038 (8)	−0.0089 (9)
C22	0.0388 (10)	0.0480 (11)	0.0541 (11)	0.0082 (8)	0.0038 (8)	0.0024 (8)
C23	0.0391 (10)	0.0543 (11)	0.0401 (9)	0.0112 (8)	0.0049 (7)	0.0072 (8)
C24	0.0412 (11)	0.0588 (12)	0.0723 (14)	0.0069 (9)	0.0105 (9)	−0.0007 (10)
C25	0.0490 (13)	0.0721 (15)	0.0875 (17)	−0.0053 (11)	0.0125 (11)	0.0076 (13)
C26	0.0368 (11)	0.0958 (18)	0.0745 (15)	0.0075 (11)	0.0149 (10)	0.0249 (13)
C27	0.0486 (13)	0.0822 (17)	0.102 (2)	0.0239 (12)	0.0258 (13)	0.0210 (14)
C28	0.0490 (12)	0.0581 (13)	0.0871 (16)	0.0151 (10)	0.0164 (11)	0.0100 (11)
O1	0.0536 (9)	0.0466 (8)	0.1322 (16)	0.0064 (7)	0.0130 (9)	−0.0068 (9)

### Geometric parameters (Å, º)

**Table:**

C1—C2	1.385 (3)	C14—C15	1.465 (3)
C1—C6	1.392 (3)	C15—C20	1.386 (3)
C1—C7	1.514 (3)	C15—C16	1.397 (3)
C2—C3	1.377 (3)	C16—C17	1.381 (3)
C2—H2	0.9300	C17—C18	1.377 (3)
C3—C4	1.369 (3)	C17—H17	0.9300
C3—H3	0.9300	C18—C19	1.377 (3)
C4—C5	1.376 (4)	C18—H18	0.9300
C4—H4	0.9300	C19—C20	1.374 (3)
C5—C6	1.377 (3)	C19—H19	0.9300
C5—H5	0.9300	C20—H20	0.9300
C6—H6	0.9300	C21—C22	1.514 (2)
C7—C21	1.526 (2)	C21—H21A	0.9700
C7—C8	1.554 (2)	C21—H21B	0.9700
C7—H7	0.9800	C22—O1	1.211 (2)
C8—C9	1.514 (3)	C22—C23	1.497 (3)
C8—C16	1.518 (3)	C23—C24	1.374 (3)
C8—H8	0.9800	C23—C28	1.389 (3)
C9—C10	1.379 (3)	C24—C25	1.384 (3)
C9—C14	1.392 (3)	C24—H24	0.9300
C10—C11	1.382 (4)	C25—C26	1.369 (3)
C10—H10	0.9300	C25—H25	0.9300
C11—C12	1.368 (4)	C26—C27	1.360 (4)
C11—H11	0.9300	C26—H26	0.9300
C12—C13	1.383 (3)	C27—C28	1.378 (3)
C12—H12	0.9300	C27—H27	0.9300
C13—C14	1.385 (3)	C28—H28	0.9300
C13—H13	0.9300		
			
C2—C1—C6	117.1 (2)	C9—C14—C15	108.76 (17)
C2—C1—C7	123.13 (17)	C20—C15—C16	120.69 (19)
C6—C1—C7	119.78 (18)	C20—C15—C14	130.71 (19)
C3—C2—C1	121.9 (2)	C16—C15—C14	108.60 (17)
C3—C2—H2	119.1	C17—C16—C15	119.75 (18)
C1—C2—H2	119.1	C17—C16—C8	130.05 (18)
C4—C3—C2	120.0 (2)	C15—C16—C8	110.12 (16)
C4—C3—H3	120.0	C18—C17—C16	119.0 (2)
C2—C3—H3	120.0	C18—C17—H17	120.5
C3—C4—C5	119.6 (2)	C16—C17—H17	120.5
C3—C4—H4	120.2	C19—C18—C17	121.2 (2)
C5—C4—H4	120.2	C19—C18—H18	119.4
C4—C5—C6	120.3 (2)	C17—C18—H18	119.4
C4—C5—H5	119.9	C20—C19—C18	120.7 (2)
C6—C5—H5	119.9	C20—C19—H19	119.7
C5—C6—C1	121.2 (2)	C18—C19—H19	119.7
C5—C6—H6	119.4	C19—C20—C15	118.7 (2)
C1—C6—H6	119.4	C19—C20—H20	120.6
C1—C7—C21	113.49 (15)	C15—C20—H20	120.6
C1—C7—C8	112.72 (15)	C22—C21—C7	113.60 (16)
C21—C7—C8	111.24 (15)	C22—C21—H21A	108.8
C1—C7—H7	106.3	C7—C21—H21A	108.8
C21—C7—H7	106.3	C22—C21—H21B	108.8
C8—C7—H7	106.3	C7—C21—H21B	108.8
C9—C8—C16	102.14 (15)	H21A—C21—H21B	107.7
C9—C8—C7	113.13 (15)	O1—C22—C23	120.33 (17)
C16—C8—C7	116.57 (15)	O1—C22—C21	120.44 (17)
C9—C8—H8	108.2	C23—C22—C21	119.22 (17)
C16—C8—H8	108.2	C24—C23—C28	118.21 (18)
C7—C8—H8	108.2	C24—C23—C22	123.47 (17)
C10—C9—C14	120.1 (2)	C28—C23—C22	118.30 (18)
C10—C9—C8	129.6 (2)	C23—C24—C25	120.8 (2)
C14—C9—C8	110.35 (17)	C23—C24—H24	119.6
C9—C10—C11	119.1 (2)	C25—C24—H24	119.6
C9—C10—H10	120.4	C26—C25—C24	120.1 (2)
C11—C10—H10	120.4	C26—C25—H25	119.9
C12—C11—C10	120.7 (2)	C24—C25—H25	119.9
C12—C11—H11	119.7	C27—C26—C25	119.7 (2)
C10—C11—H11	119.7	C27—C26—H26	120.1
C11—C12—C13	121.1 (2)	C25—C26—H26	120.1
C11—C12—H12	119.5	C26—C27—C28	120.6 (2)
C13—C12—H12	119.5	C26—C27—H27	119.7
C12—C13—C14	118.4 (2)	C28—C27—H27	119.7
C12—C13—H13	120.8	C27—C28—C23	120.5 (2)
C14—C13—H13	120.8	C27—C28—H28	119.7
C13—C14—C9	120.5 (2)	C23—C28—H28	119.7
C13—C14—C15	130.7 (2)		
			
C6—C1—C2—C3	−0.7 (3)	C13—C14—C15—C16	−179.84 (19)
C7—C1—C2—C3	177.51 (19)	C9—C14—C15—C16	1.1 (2)
C1—C2—C3—C4	0.7 (3)	C20—C15—C16—C17	−1.9 (3)
C2—C3—C4—C5	−0.1 (4)	C14—C15—C16—C17	177.38 (16)
C3—C4—C5—C6	−0.4 (4)	C20—C15—C16—C8	−179.05 (17)
C4—C5—C6—C1	0.4 (4)	C14—C15—C16—C8	0.3 (2)
C2—C1—C6—C5	0.2 (3)	C9—C8—C16—C17	−178.08 (18)
C7—C1—C6—C5	−178.1 (2)	C7—C8—C16—C17	58.1 (3)
C2—C1—C7—C21	−2.0 (2)	C9—C8—C16—C15	−1.34 (19)
C6—C1—C7—C21	176.14 (17)	C7—C8—C16—C15	−125.17 (17)
C2—C1—C7—C8	125.57 (19)	C15—C16—C17—C18	1.6 (3)
C6—C1—C7—C8	−56.3 (2)	C8—C16—C17—C18	178.06 (19)
C1—C7—C8—C9	174.43 (15)	C16—C17—C18—C19	−0.2 (3)
C21—C7—C8—C9	−56.8 (2)	C17—C18—C19—C20	−0.9 (4)
C1—C7—C8—C16	−67.6 (2)	C18—C19—C20—C15	0.6 (3)
C21—C7—C8—C16	61.2 (2)	C16—C15—C20—C19	0.8 (3)
C16—C8—C9—C10	−176.92 (19)	C14—C15—C20—C19	−178.3 (2)
C7—C8—C9—C10	−50.8 (3)	C1—C7—C21—C22	−73.5 (2)
C16—C8—C9—C14	2.01 (19)	C8—C7—C21—C22	158.13 (16)
C7—C8—C9—C14	128.12 (17)	C7—C21—C22—O1	−20.7 (3)
C14—C9—C10—C11	0.3 (3)	C7—C21—C22—C23	160.49 (16)
C8—C9—C10—C11	179.11 (19)	O1—C22—C23—C24	172.4 (2)
C9—C10—C11—C12	1.5 (3)	C21—C22—C23—C24	−8.9 (3)
C10—C11—C12—C13	−1.4 (4)	O1—C22—C23—C28	−5.8 (3)
C11—C12—C13—C14	−0.4 (3)	C21—C22—C23—C28	172.95 (18)
C12—C13—C14—C9	2.2 (3)	C28—C23—C24—C25	0.6 (3)
C12—C13—C14—C15	−176.83 (19)	C22—C23—C24—C25	−177.6 (2)
C10—C9—C14—C13	−2.1 (3)	C23—C24—C25—C26	−0.3 (4)
C8—C9—C14—C13	178.83 (16)	C24—C25—C26—C27	−0.1 (4)
C10—C9—C14—C15	177.08 (17)	C25—C26—C27—C28	0.3 (4)
C8—C9—C14—C15	−2.0 (2)	C26—C27—C28—C23	0.0 (4)
C13—C14—C15—C20	−0.6 (3)	C24—C23—C28—C27	−0.4 (3)
C9—C14—C15—C20	−179.7 (2)	C22—C23—C28—C27	177.9 (2)
